# Multivessel spontaneous coronary artery dissection in a young woman using cabergoline

**DOI:** 10.1530/EDM-24-0129

**Published:** 2026-06-18

**Authors:** Rachel Byrne, Paul Shiels, Rajesh Kumar

**Affiliations:** ^1^ Midland Regional Hospital, Tullamore, County Offaly, Ireland; ^2^ St James’s Hospital, County Dublin, Ireland

**Keywords:** pituitary, cardiovascular, reproduction

## Abstract

**Summary:**

We present a unique case of multivessel spontaneous coronary artery dissection (SCAD) in a young woman using cabergoline. This woman presented with an acute coronary syndrome (ACS) on a background of cabergoline use for a prolactinoma diagnosed over 20 years ago. Coronary angiogram revealed multivessel coronary artery dissection in the absence of occlusive disease. SCAD is an increasingly recognised cause of acute ACS; however, cabergoline-induced SCAD is exceedingly rare with limited literature to date. We highlight this important clinical entity to physicians, who must have a high index of clinical suspicion of this diagnosis in ACS presentations on a background of cabergoline use.

**Learning points:**

- We need a high index of clinical suspicion of SCAD in young women with ACS presentations.
- Cabergoline therapy may be an important risk factor for consideration in those presenting with a possible SCAD.

## Background

Spontaneous coronary artery dissection (SCAD) is a unique clinical entity gaining rapid recognition as an important cause of myocardial infarction. SCAD is defined as a spontaneous separation of the coronary arterial wall, resulting in an accumulation of blood in a formed false lumen. This leads to compression of the true lumen and subsequent ischaemia to the myocardium and ACS. Modern definitions of SCAD exclude those which are related to trauma, iatrogenic in nature or secondary to isolated atherosclerotic coronary disease (1). Predisposing exposures include female gender, pregnancy, exposure to female hormones, fibromuscular dysplasia, coronary vasospasm, physical/emotional triggers and pharmacological agents, such as cocaine- or ergot-derived dopamine agonists (2).

**Table 1:** Summary of patient’s cardiovascular risk factors.

Parameters	Values	NR
Lipids		
Total cholesterol, mmol/L	4.5	2.5–5
HDL, mmol/L	1.63	1–2
LDL, mmol/L	2.71	0–3
HBA1c, mmol/mol	33	20–42
Glucose, mmol	5.2	
BMI	In the normal range	
BP, mmHg	100/67	
Tobacco	Never smoker	
Physical activity	Moderate activity levels	
Diet	Healthy, balanced diet	

NR, normal range.

Cabergoline is an ergot-derived dopamine receptor agonist, which is predominantly used to treat hyperprolactinaemia secondary to a prolactinoma. Prolactinomas are the most common subtype of pituitary tumour and usually present as galactorrhoea, menstrual irregularities or subfertility. Our patient is no exception to this, having presented in a similar way upon her diagnosis of prolactinoma over 20 years prior. Today we present a unique case of a young woman with multivessel SCAD on a background of cabergoline use. To date there is limited literature available on cabergoline-induced SCAD, and we describe the first ever reported case in our country.

## Case presentation

In early 2024 a 45-year-old lady presented to the Emergency Department with an acute onset of severe central chest pain of 30-min duration, which was heavy in nature, radiated to the arms and back while driving and was associated with nausea, causing her to vomit once. She denied any other associated symptoms. Her admission blood pressure was 100/67 mmHg, heart rate was 61 beats/min, respiratory rate was 14 breaths/min, oxygen saturations were 98% on room air, and she was apyrexial. Her physical exam was normal without any phenotypic features of connective tissue disorders, pregnancy test was negative, and she denied any recent stress or systemic illness.

She has a background of hypercholesterolaemia and an irregular menstrual cycle, which ultimately led to her diagnosis with a prolactinoma 20 years prior. Her regular medication included cabergoline 500 μg weekly, which she had been taking regularly since diagnosis without any endocrinology follow-up, and atorvastatin 40 mg once a day. She had a regular menstrual cycle, did not have any features of perimenopause and was not taking any hormonal medications or supplements. There was no family history of any cardiovascular or genetic conditions. See Table 1 for a summary of her cardiovascular risks.

## Investigations

**Figure 1:**
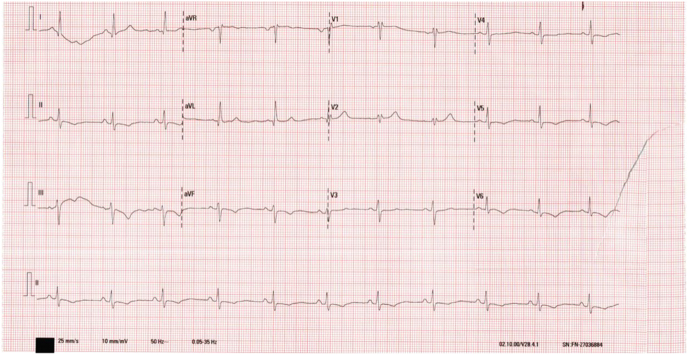
Present ECG showing T-wave inversion in lead II, III, AVF, V5 and V6.

Her presenting ECG confirmed T-wave inversion in lead II, III, AVF, V5 and V6 (Fig. 1).

Her initial troponin was high at 671 ng/L (NR: 0–14 ng/L). The remainder of her bloods including a full blood count, liver profile, renal profile and bone profile were normal. Her CRP was 1.2 mg/L (NR: 0–5 mg/L). She was transferred to our coronary care unit for serial data and was started on non-ST-segment elevation myocardial infarction (NSTEMI) management.

**Figure 2:**
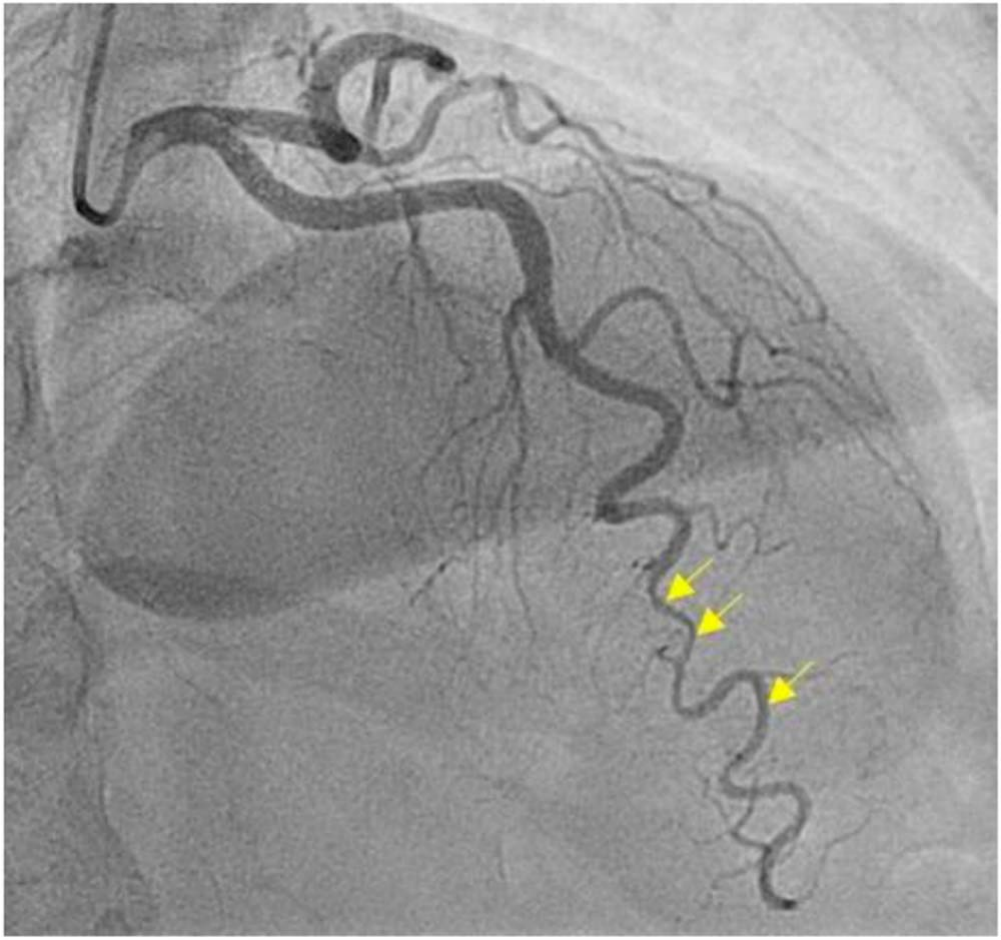
LAD artery dissection.

**Figure 3:**
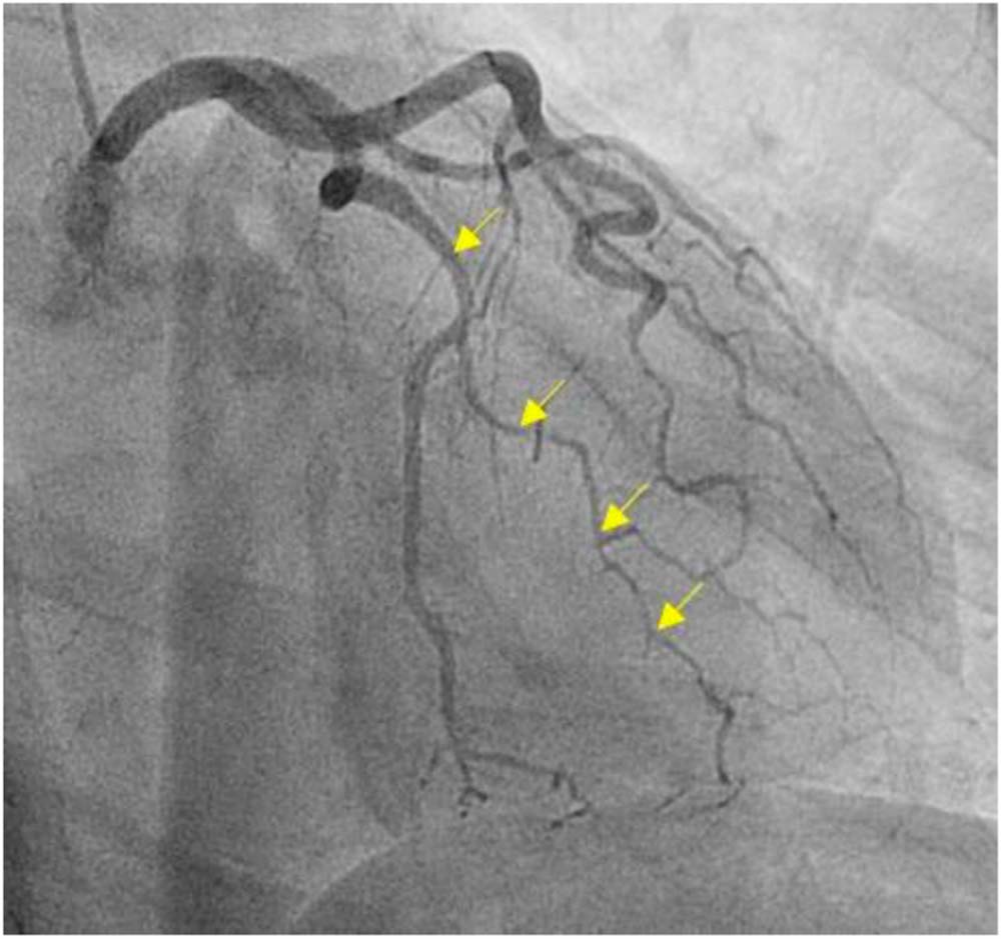
Left circumflex artery dissection.

Echocardiogram confirmed posterior-lateral and apical hypokinesis with impaired left ventricular ejection fraction 45%. She was transferred to our local primary percutaneous coronary intervention (PCI) centre where diagnostic coronary angiogram revealed a mid to distal left anterior descending (LAD) artery SCAD with patent distal vessel flow (Fig. 2). The left circumflex artery also showed SCAD extending all the way to the distal end of obtuse marginal branch (Fig. 3). The right coronary artery was normal.

Computerised tomographic angiogram of the aorta, carotids and renal arteries was normal. Cardiac magnetic resonance imaging with stress perfusion was reassuring, confirming SCAD with no fibrosis and a structurally normal heart. Her serum connective tissue screening and vasculitis screens were all negative. The absence of any clinical features of connective tissue diseases on examination, ECG, CT or on echo meant genetic testing is not recommended (3). Prolactin level two months later and off cabergoline for the same time was 663 (NR: 0–999). MRI pituitary was performed two years later due to a delay in access to outpatient MRI. This showed a partially empty sella filled with CSF and a pituitary thickness of 3 mm. The anterior and posterior pituitaries are normal in size without evidence of a lesion.

## Treatment, outcome and follow-up

She was managed conservatively based on the latest European and American SCAD guidelines, given that she was haemodynamically stable with no obstructive flow features on coronary angiography. She remained clinically stable without any further complaints during her hospitalisation. Her ECG normalised during her stay. She recovered with no further episodes of chest pain and her serial troponins declined from 671 > 230 > 185 > 100 ng/L. She was started on guideline-directed medical therapy with discontinuation of her cabergoline after consultation with endocrinology. She was discharged with outpatient cardiology and endocrine follow-up in place. She was followed up at 6 weeks with normalisation of her left ventricular function and outpatient CT coronary angiogram 6 months later confirmed complete healing of her coronary arteries.

## Discussion

SCAD was once thought to be a rare entity; however, SCAD prevalence has increased secondary to the growing use of early diagnostic angiography and intracoronary imaging (1). The estimated prevalence of SCAD in ACS patients ranges between 1.7 and 4% across the globe with incidence increasing to 43% in peripartum ACS women (4). Since its first description on autopsy in 1931, there has been a surge in the diagnosis of SCAD secondary to raised awareness, increased publication, attention through social media and improved recognition (4).

Diagnosis of SCAD is heavily reliant on clinicians having a high index of suspicion. The most common presentation is a troponin-positive ACS with or without ST changes on ECG. These patients are often young females, and in the absence of any significant cardiac risk factors, they may easily be missed. Once SCAD is suspected, coronary angiography is the recommended gold standard approach. However, angiography has its own limitations: it is a two-dimensional view of a three-dimensional structure (5). Dedicated use of intracoronary imaging (optical coherence tomography and intravascular ultrasound) leads to better diagnosis of SCAD, but it is associated with additional risk and cost as well as being not widely available (5). Angiography also provides the opportunity for early intervention in selected severe cases. Conventional angiographic description of SCAD consists of lumen narrowing, dual lumen visualisation, spiral dissections and extraluminal contrast straining with filling defects. The Saw angiographic SCAD classification criteria have been widely accepted and utilised in the recognition of SCAD (6).

Optimal management of SCAD is undetermined. Given that SCAD is a relatively new clinical entity, there is limited data available on the best approach to management. There are no randomised control trials like atherosclerotic ACS comparing medical therapies with revascularisation approaches. Current management offered by the American Heart Association (5) and the European Society of Cardiology (3) recommends conservative medical management in the first instance. This is due to the increased risk of iatrogenic extension of the arterial dissection and potential occlusion of the true lumen by catheter interventions. Evidence from observational studies and case series suggest on follow-up angiography that majority of SCAD will heal spontaneously with conservative management (5). Conservative management with beta-blocker therapy has been shown to reduce recurrence rates by reducing shearing stress on arterial wall (3). ACE inhibitors are recommended for those with left ventricular dysfunction similar to any other ACS; however, they are not studied solely in this population yet (5). There is a controversial role for statins in the management of SCAD, and some studies even suggested a higher reoccurrence with their use. Given the aetiology is not due to atherosclerotic plaque rupture, unless lipid levels are found to be raised, statins are not recommended routinely (5). Finally, the research is inconclusive on the use of antiplatelets and anticoagulants, with no studies yet published comparing dual antiplatelets vs aspirin alone. Most experts recommend a case-by-case consideration, with usual practice being dual antiplatelets or aspirin only use for at least 1 year in those without other bleeding risks (3, 5). Coronary artery bypass grafting or PCI is recommended in those more serious cases with ongoing ischaemia, haemodynamic instability or left main artery involvement (5).

As mentioned, there are a number of risks associated with SCAD development, including pregnancy, states of high female hormones, fibromuscular dysplasia, vasospasm, emotional triggers and pharmacological agents, such as cocaine- or ergot-derived dopamine receptor agonists such as cabergoline (2). The relationship between cabergoline and SCAD has been previously described in a small number of case reports (7, 8, 9, 10). There are many case reports of the ergot-derived dopamine receptor agonist bromocriptine, resulting in post-partum MI; however, this is due to bromocriptine-induced coronary vasospasm (BICS) rather than dissection. To our knowledge, there are no reported cases of bromocriptine-induced SCAD. There is one case report on pergolide (another ergot-derived dopamine receptor agonist)-induced SCAD; however, this was in the context of Parkinson’s disease treatment (11). Ergot-derived dopamine agonists have been reported to cause severe vasospasm, although it is more commonly associated with fibrotic valvulopathies (12). Cabergoline-induced SCAD may be due to the aforementioned vasospasm leading to loss of normal vessel elasticity and compliance, resulting in a subsequent tear. Another proposed mechanism is related to the high hormonal state induced by the primary tumour.

## Declaration of interest

The authors declare that there is no conflict of interest that could be perceived as prejudicing the impartiality of the research reported.

## Funding

This research did not receive any specific grant from any funding agency in the public, commercial or not-for-profit sector.

## Patient consent

Written informed consent for publication of their clinical details and/or clinical images was obtained from the patient.

## Author contribution statement

RB, PS and RK were all directly involved in this patient’s care. They all contributed to the writing of this article, and all gave final approval over this submission.

## References

[1] Saw J, Mancini GBJ & Humphries KH. Contemporary review on spontaneous coronary artery dissection. J Am Coll Cardiol 2016 68 297–312. (10.1016/j.jacc.2016.05.034) 27417009

[2] Lewey J, El Hajj SC & Hayes SN. Spontaneous coronary artery dissection: new insights into this not-so-rare condition. Annu Rev Med 2022 73 339–354. (10.1146/annurev-med-052819-023826) 35084994

[3] Adlam D, Alfonso F, Maas A, et al. European Society of Cardiology, acute cardiovascular care association, SCAD study group: a position paper on spontaneous coronary artery dissection. Eur Heart J 2018 39 3353–3368. (10.1093/eurheartj/ehy080) 29481627 PMC6148526

[4] Djokovic A, Krljanac G, Matic P, et al. Pathophysiology of spontaneous coronary artery dissection: hematoma, not thrombus. Front Cardiovasc Med 2023 10 1260478. (10.3389/fcvm.2023.1260478) 37928766 PMC10623160

[5] Hayes SN, Kim ESH, Saw J, et al. Spontaneous coronary artery dissection: current state of the science: a scientific statement from the American Heart Association. Circulation 2018 137 e523–e557. (10.1161/cir.0000000000000564) 29472380 PMC5957087

[6] Saw J, Mancini GB, Humphries K, et al. Angiographic appearance of spontaneous coronary artery dissection with intramural hematoma proven on intracoronary imaging. Catheter Cardiovasc Interv 2016 87 54–61. (10.1002/ccd.26022) 26198289

[7] Mehta NK, Malkani S & Ockene I. Spontaneous coronary artery dissection during cabergoline therapy. Tex Heart Inst J 2012 39 92–94. 22412238 PMC3298899

[8] Ikeda S, Le J, Verma S, et al. A novel case of spontaneous coronary artery dissection during cabergoline therapy for prolactinoma. JACC Case Rep 2020 2 1684–1687. (10.1016/j.jaccas.2020.07.024) 34317034 PMC8312105

[9] Saleh Z, Koshy S, Sidhu V, et al. Spontaneous coronary artery dissection in association with cabergoline therapy. BMJ Case Rep 2021 14 240022. (10.1136/bcr-2020-240022) PMC7875262 33563672

[10] Ruhela MR, Ola RK & Bagarhatta R. Acute coronary syndrome in a young female: a rare occurrence with cabergoline. Int J Adv Res 2021 10 640–643. (10.21474/IJAR01/14084)

[11] Glendenhuys A, Mourant T, Johnston T, et al. Pergolide and coronary artery dissection. Br J Cardiol 2002 9 AIC 32.

[12] Al-Zubaidi AS & Afandi B. Severe digital vasospasm caused by cabergoline. Saudi Med J 2005 26 1153–1155. (10.15537/1658-3175.3058) 16047079

